# Factors Impacting Intent to Share Multigenic Cancer Testing Results in a Community Hospital Setting

**DOI:** 10.3390/jpm14090987

**Published:** 2024-09-17

**Authors:** Wamia Siddiqui, Joel E. Pacyna, Sean M. Phelan, Jeremy C. Jones, N. Jewel Samadder, Richard R. Sharp

**Affiliations:** 1 Biomedical Ethics Research Program, Mayo Clinic, Rochester, MN 55905, USA; 2 Division of Healthcare Delivery Research, Mayo Clinic, Rochester, MN 55905, USA; 3 Division of Hematology and Oncology, Mayo Clinic, Jacksonville, FL 32224, USA; 4 Division of Gastroenterology and Hepatology, Department of Medicine, Mayo Clinic, Phoenix, AZ 85054, USA; 5 Department of Clinical Genomics, Mayo Clinic, Phoenix, AZ 85054, USA; 6 Center for Individualized Medicine, Mayo Clinic, Phoenix, AZ 85054, USA; 7 Department of Quantitative Health Sciences, Mayo Clinic, Rochester, MN 55905, USA

**Keywords:** multigenic panel testing, health disparities, genetic counseling, family communication, cascade screening

## Abstract

Background/Objectives: Multi-gene, multi-cancer, hereditary cancer risk screenings may be useful in cancer prevention and treatment, not only for cancer patients but also for patients’ family members. If genetic cancer screening is to have the widest possible benefit, it must be extended into diverse cancer care settings that serve diverse patient communities, providing cancer patients and their relatives with individualized cancer risk evaluations. Little research, to date, has examined the impact of extending multigenic cancer screening into diverse settings. Without empirical data characterizing the support needs of cancer patients and their family members, we may not adequately satisfy the needs of all patients and risk exacerbating existing disparities in cancer care and outcomes. Methods: We examined patient perspectives on the sharing of genetic results with at-risk family members by surveying a racially diverse sample of cancer patients receiving a multi-gene, multi-cancer risk screen in a community hospital setting. Results: In a survey of 230 cancer patients, we found that intent to share results with family members was high but varied across family member types. More respondents planned to disclose results to at least one sister (82.5%) compared to at least one brother (73.1%). Over one-fourth of participants (27.4%) were either uncertain about sharing or intended to withhold their genomic screening results from at least one at-risk family member eligible for cascade testing. Participants were more likely to withhold their results from a sibling than from a child. Furthermore, intent to share across all family member types was lower if probands failed to identify at least one benefit to sharing. Conclusions: Understanding factors associated with decisions to share results with at-risk relatives in diverse patient populations can help clinicians support cascade genetic cancer screenings in diverse communities and settings.

## 1. Introduction

Multi-gene, multi-cancer, hereditary risk screening (multigenic cancer screening) is being used in the clinical care of cancer patients with increasing frequency. Although this is partly due to improvements in affordability and the accuracy of multigenic cancer screenings [1], the increased usage is also due to the high-value opportunities it provides for identifying families with higher-than-typical heritable cancer risk. In a recent study conducted at Mayo Clinic, 15% of all cancer patients who received multigenic cancer screening tested positive for a heritable cancer mutation (sometimes, but not always, related to their current cancer diagnosis) [2]. This positivity rate suggests a high utility for multigenic cancer screenings in cancer patients through the identification of candidates for cascade screenings to identify heritable cancer risk [2,3,4,5,6,7,8,9]. In many cases, early identification of cancer risks supports increased cancer screening measures (e.g., increased mammography and colonoscopy) and, in some cases, preventive interventions in at-risk family members [2,4,9,10,11].

In order for multigenic cancer screening to achieve its widest benefit, two things must occur. First, multigenic cancer screenings must be extended into more diverse cancer care settings that reach patients not otherwise served by large academic medical centers. Second, within these settings, genetic relatives of cancer patients with positive screens must be informed that their family member carries a heritable cancer risk and that additional screening is recommended (familial cascade testing). To date, very little research has examined either of these factors; we know little about the impact of extending multigenic cancer screenings into diverse settings and little about the dissemination of cancer risk information in these diverse settings within genetically related families [12]. Furthermore, low-resource settings often serve significant numbers of minority and historically underserved populations [13,14,15,16] and may not have pre-existing infrastructure in place to support patients through the process of receiving genetic evaluation, disseminating results to their family members, and participating in additional therapeutic or preventive interventions indicated by a positive test. In the absence of understanding patients’ support needs, and consequently failing to tailor clinical interventions to support effective results dissemination, we risk further widening existing disparities in cancer care.

To begin addressing this knowledge gap, we conducted a survey in the context of a multigenic cancer screening study conducted at St. Vincent’s Medical Center in Jacksonville, Florida, a faith-based, non-profit health system that focuses on vulnerable groups within the surrounding community. Our aim was to better understand the views of cancer patients in these settings with respect to disseminating multigenic cancer screening results to their family members.

## 2. Methods

### 2.1. Participants

We surveyed cancer patients who were enrolled in the INHERIT Study: A Trial of Proactive Genetic Testing in Cancer Patients at St. Vincent’s Mayo Clinic Embedded Cancer Center. All participants had been recruited from the Mayo Clinic Cancer Center at St. Vincent’s Medical Center Riverside, in Jacksonville, Florida, in person, at scheduled cancer appointments. St. Vincent’s Medical Center is part of the larger Ascension Healthcare System, a faith-based, non-profit hospital system and the largest Catholic health system in the United States, with an emphasis on service to vulnerable groups within the surrounding community [17]. Participants were at least 18 years of age and had an active solid tumor cancer diagnosis, including, but not limited to, gastrointestinal, breast, gynecological, genitourinary, skin, CNS/brain, head/neck, musculoskeletal, or cancer of unknown primary origin. Participants with an active hematologic malignancy or known history of multigenic cancer testing at either the Mayo Clinic or St. Vincent’s Healthcare in the past two years were excluded. No selection was made based on cancer stage, family history of cancer, or date of diagnosis.

### 2.2. Study Procedures

Multigenic cancer testing was provided to each participant using a commercially available, 84 cancer gene panel, the “Multi-Cancers Genetic Panel” (Invitae, San Francisco, CA, USA). All participants viewed a pre-test genetic counseling video informing patients about hereditary cancer and genomic screening, without highlighting either benefits or barriers to sharing the results with family. Participants’ physicians and clinical teams were informed of the results regardless of the outcomes. All participants were scheduled for a phone consultation with a genetic counselor to discuss the results. Any participants found to have a pathogenic variant were offered no-cost site-specific cancer genetic testing for at-risk relatives within three months of receiving the result. Once confirmed that patients had spoken with either a genetic counselor or an oncologist about their test results, paper documentation was sent to participants’ houses via mail and uploaded to an electronic patient portal for viewing.

### 2.3. Survey

Participants completed a brief, multi-part, 60-item survey after having their blood drawn for the genetic testing and prior to receiving their results. The survey examined participants’ intent to share their test results across types of family members, anticipated barriers and benefits to sharing, reflections on family communication and social support, views towards their medical care received, attitudes towards their healthcare experiences, and various metrics surrounding hereditary cancer, such as placement of blame and motivations to learn results. Demographic information and socioeconomic variables (including age, sex, ethnicity, race, health insurance status, family history of cancer, and site of initial cancer diagnosis) were abstracted from patients’ electronic health records.

### 2.4. Data Collection and Analysis

Data collection for the survey occurred from June 2020 to September 2021. Data analysis was performed using R Statistical Software (R Foundation for Statistical Computing, Vienna, Austria, version 4.3.2). Descriptive statistics and comparative analyses utilized Chi-Square tests and Fischer’s exact tests where appropriate. Participants for whom specific family member types were not applicable, such as in circumstances where a proband did not have a living parent or did not have any siblings, were excluded from the analysis of intent to share. For this analysis, spouses were not considered to be at-risk relatives.

## 3. Results

Out of 230 individuals who consented to participate in the INHERIT study, we received 219 completed surveys (95.2% completion rate). Table 1 provides demographic characteristics of the 219 survey respondents. A majority were female (78.1%), and the median age was 63 years. One-third (33.3%) of respondents were Black or African American; 39.6% of respondents were non-white. In total, 5.5% of respondents were Hispanic or Latino. A majority of survey respondents received public health insurance, through either Medicaid or Medicare (52.6%), or through government-sponsored military health insurance/Tricare (5.1%). In total, 40% of respondents were enrolled in commercial insurance, and 2.3% of respondents were uninsured. A majority of respondents had been diagnosed with breast cancer (60.7%). Other cancer types included lung (10.5%) and colorectal (7.3%) cancer. More than half of the respondents (58.9%) had a known family history of cancer.

A majority of respondents indicated an intent to share their multigenic cancer screening results with all living genetic relative types (Table 2). One in four respondents indicated they were unsure about sharing or did not plan to share their results with at least one family member type. Respondents’ plans to share with all male relative types compared to all female relative types were not significant. However, participants were more likely not to share results with at least one parent than with one sibling (41.4% vs. 23.2%, *p* = 0.002). Furthermore, respondents were more likely to withhold results from at least one sibling than from one or more children (23.2% vs. 14.1%, *p* = 0.031).

Table 3 compares the responses of participants planning to share their test results with all extant relative types, with those who may withhold results from one or more relative types, to questions related to the benefits and barriers to sharing multigenic cancer screening results with genetic relatives. Participants who intended to share with all extant relative types were significantly more likely to identify benefits to sharing their genetic test results across the three metrics listed on the survey (*p* = <0.001), with the most cited benefits including family members responding positively and facilitating family members decisions about their healthcare. Twenty-three percent (47 of 209 respondents) identified at least one barrier to sharing their genetic testing results with their family members. Probands in this sample who did not recognize any benefits to testing were not significantly more likely to identify barriers to genetic testing. As shown in Table 4, identifying at least one benefit of sharing was a strong predictor of intent to share test results across all family member types.

## 4. Discussion

Multi-gene, multi-cancer, hereditary cancer risk screening is becoming more common in cancer care because of its value to both probands and their family members. To avoid the potential for multigenic cancer screening to exacerbate inequities in cancer care, it is critical to examine its application in clinical settings that serve diverse populations without the support structures found in academic medical centers. Our study is one of the first to examine participants’ intent to share multigenic cancer screening results in the context of a community hospital setting. Our findings highlight three factors for clinicians and health systems leaders to consider as multigenic cancer screening is extended into more diverse settings.

First, a strong majority of participants intended to share their results across all family member types. This finding aligns with our previous work, which investigated predominantly white, well-educated, and older survey participants receiving care at an academic medical center [18,19]. Likewise, the proportion of respondents indicating that they were not planning to share their results (or who indicated that they were “unsure” about whether to share their results) varied across family member types. This finding aligns with trends observed in prior studies, which found that probands’ intention to share with their parents tended to be lower than with their adult children [18,20]. While 88.3% of applicable respondents in our study planned to share their results with at least one adult daughter and 85.0% with at least one adult son, only 49.1% of our respondents intended to share with their fathers and 64.5% with their mothers.

The median age of participants in our study was 63 years, and over half of all participants reported a known family history of cancer. Survey participants may have been less likely to share results with parents due to previous cancer diagnoses or assumptions about susceptibility to cancer due to advanced age. These patterns are consistent with prior studies of intent to share within other populations, suggesting that probands in different contexts may undergo similar decision-making processes when it comes to sharing genetic results with relatives, often weighing and balancing concerns surrounding family members’ reactions to the new information and potential worrying [18,20].

Second, although there were no statistical differences between plans to withhold results from one or more male family members vs. from one or more female family members, sharing intentions trended higher with female family members who were siblings or parents. A smaller percentage of respondents planned to share their genomic screening results with their fathers and brothers compared to their mothers and sisters, respectively. This trend was not seen in adult daughters compared to sons. Studies have found that Black women with breast cancer diagnoses may have reduced disclosure of health information with their male relatives [21]. Given the high representation of participants with breast cancer diagnoses, this indicates that probands may mistakenly associate multigenic panel testing, which screens for 80+ variants, with their current breast cancer diagnoses, believing that male relatives face lower susceptibility to all of the pathogenic cancer variants tested for compared to their female counterparts. Male family members are both less likely to be informed of familial pathogenic variants, and if informed, to subsequently pursue cascade testing [9,22,23]. This elucidates a potentially important misconception and stresses the need to highlight the relevance of multigenic screening results for all family members who may be impacted, regardless of their gender.

Third, more than a quarter of participants (27.5%) had at least one at-risk family member from whom they intended to withhold their genetic test results. Participants who planned to share their genetic test results were significantly more likely to identify benefits to sharing results, though both groups shared similar perspectives on anticipated barriers. These data suggest that failure to recognize the benefits of hereditary cancer screening results may be an important predictor of intent to withhold testing results from at least one at-risk relative, across all types of family members. A stronger appreciation of the potential impact of the preventative health benefits of genomic screening is imperative, as patients who did identify advantages appeared more likely to take action to improve their family members’ future health by sharing results information. Despite being informed about genetic screening for hereditary cancer via a pre-recorded video, many patients appeared not to realize that the results from multigenic panel testing, embedded within their individualized cancer care, could have implications for their relatives’ health. Our data illustrates that an appreciation of potential improvements in health outcomes for relatives is a strong motivator to share, highlighting again the need for clinicians to emphasize the dual capacity of multigenic cancer screening to impact family members’ health alongside their patients’ own cancer care [5,24].

While our survey study focused mainly on factors that may impact the sharing of genetic cancer screening results in the context of a diverse, mission-driven community hospital, our results underscore the importance of closely examining the impact of extending genetic cancer screening opportunities to patients receiving cancer care in settings that do not necessarily have the same resources or health care infrastructure as academic medical centers. At face value, efforts to extend genetic services to lower resource settings seem to address a noteworthy inequity in healthcare access. However, without adequate support for patients throughout all aspects of the process (including increasing health literacy about their results or facilitating the process of disseminating results to at-risk family members), efforts to expand access to genomic screening services may have little positive impact, or worse, may ultimately complicate the cancer care experience for patients and thereby exacerbate the very disparities they are intended to address.

Our results also highlight the importance of developing evidence-based supportive processes and interventions that enhance patient understanding and facilitate patients’ efforts to communicate their genetic results to family members. While a number of tools exist to support patients receiving genetic information [25], nearly all of them have been developed in academic medical settings and are often designed to be used in conjunction with pre-test genetic counseling. Additionally, many of these tools have been developed and validated in majority populations. Tools designed to support patients in non-academic medical settings will require validation in the populations they are intended to serve and implementation strategies tailored to the unique workflows and support structures that are available in those settings.

Future research should continue to evaluate the support needs of patients receiving genetic cancer screening in diverse settings, with the goal of designing and testing interventions to support patients as they process genetic cancer risk and disseminate information about heritable cancer risk to their at-risk relatives. Future research should also continue to explore the influence of cultural factors on how families share risk information with each other. Future research should further assess whether influential cultural factors are amenable to intervention in diverse care settings.

## 5. Limitations

There are limitations to this study. Our survey captured intent to share prior to receipt of results, and we used intent to share as a predictor of communication of results with relatives, assuming that probands’ actions after receiving results align with their initial intent. Our survey did not measure the influence of the genetic results patients received, and patients’ final sharing decisions may be dependent on whether they are informed of positive or negative results and the type of cancer risk identified. This survey also used language measuring intent to share with “at least one” brother, sister, adult son, and adult daughter, which did not allow us to examine differences in intent that might exist among individual members of each category. The relatively small sample size from a single study site may not be representative of other community hospitals. Our sample size also did not allow us to conduct demographic subgroup analyses (for example, for age, family history, or cancer site). Variations in past conversations with clinicians about hereditary cancer risk, and past experiences with cancer-related discussions may impact patients’ current approaches and perceptions. Only site-specific testing for pathogenic variants was offered to at-risk family members at no cost, rather than multigenic panel testing, which may have revealed pathogenic variants different from the known familial gene carried by the proband in approximately 5% of relatives [4].

## 6. Conclusions

Family communication of patients’ screening results and subsequent cascade testing of at-risk relatives are both pivotal in elucidating hereditary cancer risk beyond individual patients receiving multigenic cancer screening as part of their cancer care. As precision medicine tools expand, there is a pressing need to examine health disparities encountered in under-resourced healthcare settings such as smaller community hospitals. Healthcare professionals can play a crucial role in bolstering appreciation of the benefits of multigenic cancer screenings by ensuring that patients are adequately supported in the process, including facilitating the dissemination of cancer screening results to patients’ at-risk family members. To provide the highest standard of care to all cancer patients, precision medicine technologies like multigenic cancer screening must adapt to meet the needs of patients in a variety of clinical settings.

## Figures and Tables

**Table 1:** Demographic characteristics of 219 oncology patients receiving care in a community hospital setting.

	N (%)
**Age in Years**	
Mean (SD)	61.7 (11.5)
Median (Range)	63 (27–83)
**Gender**	
Female	171 (78.1)
Male	48 (21.9)
**Race**	
White	130 (59.4)
Black or African American	73 (33.3)
Asian or Pacific Islander	6 (2.7)
Other/Undisclosed	10 (4.6)
**Ethnicity**	
Hispanic or Latino	12 (5.5)
Not Hispanic or Latino	202 (92.2)
Unknown/Undisclosed	5 (2.3)
**Health Insurance Status**	
Commercial	86 (40.0)
Medicaid/Medicare	113 (52.6)
Military/Tricare	11 (5.1)
Uninsured	5 (2.3)
**Family History of Cancer**	
Yes	126 (58.9)
No	88 (41.1)
**Site of Initial Diagnosis**	
Breast	133 (60.7)
Lung	23 (10.5)
Colorectal	16 (7.3)
Melanoma	10 (4.6)
Pancreas	5 (2.3)
Prostate	5 (2.3)
Other	5 (2.3)
Esophageal	4 (1.8)
Gastric	4 (1.8)
Ovarian	3 (1.4)
Renal	3 (1.4)
Head/neck	2 (0.9)
Hepatocellular	2 (0.9)
Small bowel	2 (0.9)
Appendix	1 (0.5)
Thyroid	1 (0.5)

**Table 2:** Comparison of plans to share germline cancer screening results across family member types.

		Sharing Intention		
	Total ^b^	Plan to Share with All Members of the Family Relationship Type	May Not Share with One or More Members of the Family Relationship Type	
	N	Row % (N)	Row % (N)	*p*-Value
Survey respondents with one or more of the following living family relationships ^a^				
Any family member	212	72.6 (154)	27.4 (58)	-
Female family member(s) vs.	198	79.8 (158)	20.2 (40)	0.181
Male family member(s)	189	74.1 (140)	25.9 (49)	
Child(ren) vs.	170	85.9 (146)	14.1 (24)	0.031
Sibling(s) vs.	177	76.8 (136)	23.2 (41)	0.002
Parent(s)	87	58.6 (51)	41.4 (36)	

^a^ Blood relatives; spouses/partners not included; ^b^ Total study participants having family member type.

**Table 3:** Patient perceptions of benefits and barriers to sharing germline cancer screening results with blood relatives.

	Sharing Intention			
	Planning to Share with All Relative Types (N = 154)	May Not Share with at Least One Relative Type (N = 58)	Overall (N = 212)	
	N (%)	N (%)	N (%)	*p*-Value
**Identification of Benefits**				
My family will be glad I shared my genetic test results with them				<0.001
Yes	135 (88.2)	32 (56.1)	167 (79.5)	
No	18 (11.8)	25 (43.9)	43 (20.5)	
Sharing my genetic test results will bring my family closer together				<0.001
Yes	76 (49.7)	11 (19.3)	87 (41.4)	
No	77 (50.3)	46 (80.7)	123 (58.6)	
Sharing my genetic test results will help my family make decisions about their healthcare				<0.001
Yes	133 (86.9)	35 (61.4)	168 (80.0)	
No	20 (13.1)	22 (38.6)	42 (20.0)	
**Identification of Barriers**				
Some of my family members…				
… will be difficult to reach				0.648
Yes	13 (8.5)	6 (10.5)	19 (9.0)	
No	140 (91.5)	51 (89.5)	191 (91.0)	
… will struggle to understand my genetic test results				0.447
Yes	17 (11.3)	4 (7.0)	21 (10.1)	
No	133 (88.7)	53 (93.0)	186 (89.9)	
… will be upset with me when they hear about my genetic test results				0.013
Yes	3 (2.0)	6 (10.5)	9 (4.3)	
No	150 (98.0)	51 (89.5)	201 (95.7)	
… will not want to hear about my genetic test results				0.750
Yes	9 (5.9)	4 (7.1)	13 (6.2)	
No	144 (94.1)	52 (92.9)	196 (93.8)	
… are struggling with other personal issues and sharing my genetic test results with them will add to their problems				0.350
Yes	8 (5.3)	5 (8.8)	13 (6.2)	
No	144 (94.7)	52 (91.2)	196 (93.8)	

**Table 4:** Associations of identifying benefits of sharing results with plans to share results with family.

	Proband Identifies at Least One Benefit of Sharing Results with Family			
	Yes (N = 189)	No (N = 26)	Total	*p*-Value
Proband intends to share with…	N (%)	N (%)	N (%)	
Spouse or partner				**
Yes	114 (91.2)	14 (63.6)	128 (87.1)	
No	11 (8.8)	8 (36.4)	19 (12.9)	
Father				*
Yes	26 (60.5)	2 (14.3)	28 (49.1)	
No	17 (39.5)	12 (85.7)	29 (50.9)	
Mother				*
Yes	45 (73.8)	4 (26.7)	49 (64.5)	
No	16 (26.2)	11 (73.3)	27 (35.5)	
At least one brother				**
Yes	90 (79.6)	8 (40.0)	98 (73.7)	
No	23 (20.4)	12 (60.0)	35 (26.3)	
At least one sister				**
Yes	106 (87.6)	12 (57.1)	118 (83.1)	
No	15 (12.4)	9 (42.9)	24 (16.9)	
At least one adult son				**
Yes	104 (90.4)	9 (50.0)	113 (85.0)	
No	11 (9.6)	9 (50.0)	20 (15.0)	
At least one adult daughter				*
Yes	106 (91.4)	15 (71.4)	121 (88.3)	
No	10 (8.6)	6 (28.6)	16 (11.7)	

* *p* < 0.01. ** *p* < 0.001.

## Data Availability

The datasets presented in this article are not readily available. Requests to access the datasets should be directed to the corresponding author, Richard R. Sharp.
